# Perinatal Health Outcomes Across Rural and Nonrural Counties Within a Single Health System Catchment

**DOI:** 10.1089/whr.2022.0061

**Published:** 2023-04-17

**Authors:** Dominick J. Lemas, Claire Layton, Hailey Ballard, Ke Xu, John C. Smulian, Matthew Gurka, Matthew Shane Loop, Erica L. Smith, Callie F. Reeder, Adetola Louis-Jacques, Chu J. Hsiao, Nicole Cacho, Jaclyn Hall

**Affiliations:** ^1^ Department of Health Outcomes and Biomedical Informatics, College of Medicine, University of Florida, Gainesville, Florida, USA.; ^2^ Department of Obstetrics and Gynecology, College of Medicine, University of Florida, Gainesville, Florida, USA.; ^3^ Division of Pharmacotherapy and Experimental Therapeutics, Eshelman School of Pharmacy, University of North Carolina at Chapel Hill, Chapel Hill, North Carolina, USA.; ^4^ Department of Anthropology, College of Liberal Arts and Sciences, University of Florida, Gainesville, Florida, USA.; ^5^ Department of Pediatrics, College of Medicine, University of Florida, Gainesville, Florida, USA.

**Keywords:** County Health Rankings, FLHealthCHARTS.gov, catchment area, north central Florida, electronic health records, breastfeeding, maternal smoking

## Abstract

**Background:**

Perinatal health outcomes are influenced by a variety of socioeconomic, behavioral, and economic factors that reduce access to health services. Despite these observations, rural communities continue to face barriers, including a lack of resources and the fragmentation of health services.

**Objective:**

To evaluate patterns in health outcomes, health behaviors, socioeconomic vulnerability, and sociodemographic characteristics across rural and nonrural counties within a single health system catchment area.

**Methods:**

Socioeconomic vulnerability metrics, health care access as determined by licensed provider metrics, and behavioral data were obtained from FlHealthCHARTS.gov and the County Health Rankings. County-level birth and health data were obtained from the Florida Department of Health. The University of Florida Health Perinatal Catchment Area (UFHPCA) was defined as all Florida counties where ≥5% of all infants were delivered at Shands Hospital between June 2011 and April 2017.

**Results:**

The UFHPCA included 3 nonrural and 10 rural counties that represented more than 64,000 deliveries. Nearly 1 in 3 infants resided in a rural county, and 7 out of 13 counties did not have a licensed obstetrician gynecologist. Maternal smoking rates (range 6.8%–24.8%) were above the statewide rate (6.2%). Except for Alachua County, breastfeeding initiation rates (range 54.9%–81.4%) and access to household computing devices (range 72.8%–86.4%) were below the statewide rate (82.9% and 87.9%, respectively). Finally, we found that childhood poverty rates (range 16.3%–36.9%) were above the statewide rate (18.5%). Furthermore, risk ratios suggested negative health outcomes for residents of counties within the UFHPCA for each measure, except for infant mortality and maternal deaths, which lacked sample sizes to adequately test.

**Conclusions:**

The health burden of the UFHPCA is characterized by rural counties with increased maternal death, neonatal death, and preterm birth, as well as adverse health behaviors that included increased smoking during pregnancy and lower levels of breastfeeding relative to nonrural counties. Understanding perinatal health outcomes across a single health system has potential to not only estimate community needs but also facilitate planning of health care initiatives and interventions in rural and low-resource communities.

## Background

Adverse perinatal health outcomes, including maternal and infant mortality, are far higher in the United States compared with similarly large and wealthy countries.^1,2^ In 2017, the World Health Organization reported that the United States was one of only two countries to report a significant increase in maternal mortality since 2000.^3^ From 1990 to 2017, the maternal mortality rate in the United States has increased from 12 to 19, per 100,000 live births.^4^ Moreover, pregnant patients who did not receive prenatal care are three to five times more likely to delivery an infant of low birthweight and are five times more likely to experience infant death following delivery compared with those mothers who receive adequate prenatal care.^2^ Accumulating data demonstrates that maternal mortality is more likely among women who are residing in rural communities,^5^ are young, low-income, African American, unmarried, less educated, receive federal assistance (*i.e.,* The Special Supplemental Nutrition Program for Women, Infants, and Children), are overweight or obese before pregnancy, or who reported their pregnancy was unintended.^5,6^

Collectively, these data demonstrate that disparities in maternal and neonatal mortality exist by race/ethnicity and age in the United States,^7,8^ and recent analyses have described rural disparities as well.^9,10^

Despite significant advances in clinical care,^11–13^ there is a growing awareness that up to 90% of rural/urban differences in perinatal health outcomes are the result of nonclinical factors, known as the social determinants of health (SDoH), including social, behavioral, and economic domains.^14^ As an example, maternal education and household income^15^ are important modifiers of perinatal health behaviors such as maternal smoking^16,17^ and breastfeeding success.^18^ Maternal education across all racial groups remains the strongest factor that influences breastfeeding initiation and duration^19^ and smoking cessation success.^17^ Nearly twice as many higher-income women breastfeed exclusively at 6 months as compared with their lower-income counterparts.^20^ With respect to maternal smoking, low socioeconomic status was associated with higher risk of smoking at the time of conception and continued smoking during pregnancy.^16^ At the individual level, SDoH include factors such as education and income that occur outside of the health care provider's office and impact perinatal health outcomes along with community-level factors. Other measures that affect both perinatal health behaviors and health outcomes are childhood poverty and access to computing devices.

Women living in poverty and birthing children into poverty, are more likely to engage in risky health behaviors such as smoking during pregnancy, subsequently increasing their risk of preterm birth.^21^ Access to computing devices at the household level provides pregnant women access to telemedicine. Telemedicine has been shown to extend obstetric services beyond a single hospital, improving pregnancy outcomes and reducing medical costs.^22^ Both childhood poverty and access to telemedicine can be utilized as measure of health circumstances that influence both perinatal activities and outcomes. Research on rural perinatal health outcomes has traditionally been limited by the diverse demographics of these regions by carefully considering the distinct characteristics, inequities, and stressors occurring in rural communities.^23^

Disparities in perinatal morbidity and mortality between urban and rural residents are well documented and have been referred to as the “rural mortality penalty”.^24^ We defined and examined a perinatal health catchment area for University of Florida (UF) Health Shands Hospital, a single health care system with broad regional reach in north central Florida, to identify opportunities to improve health related to social determinants. Defining a geographical area around a health care service point is important to not only estimate community needs but also facilitate planning of health care initiatives.^25^ We characterized county-level patterns in perinatal health outcomes and behaviors, socioeconomic vulnerability, and the availability of health care providers across rural and nonrural Florida counties within this single health system catchment. The long-term goal of the University of Florida Health Perinatal Catchment Area (UFHPCA) is to facilitate collaborations in which researchers, providers, public health practitioners, and nonprofit organizations can utilize the data to develop or expand applied perinatal research, planning, and implementation, with an emphasis on improving local health outcomes.

## Data and Methods

### Perinatal catchment definition

The UFHPCA was defined at the county level from births occurring at UF Health Shands Hospital. We obtained electronic health records (EHR) from UF Health Shands Hospital that included all live births occurring between June 2011 and April 2017.^26^ In June 2011, UF Health Shands Hospital adopted a new electronic medical records system, thus we defined June 2011 as the start date of our dataset to obtain the most complete and consistent data available. At the time of this article's data collection, April 2017 was the latest data available to the researchers.

Linkage of maternal–infant records to obtain mother's residential 5- or 9-digit zip code was completed by an honest broker within the UF Health Integrated Data Repository before releasing the de-identified data to the research team. Zip codes from the delivery encounters were available on 99.1% of records. Nine-digit and most 5-digit zip codes were linked to counties. The number of births from 5-digit zip codes that cross county borders were aggregated to the county level using the 2010 Zip Code Tabulation Area to County Relationship File from the U.S. Census Bureau.^27^

UF Health EHR birth counts per county were compared with county birth counts from the Florida Bureau of Vital Statistics during the same period (June 2011–April 2017). We defined the UFHPCA as all counties where 5% or more of county births occurred at UF Health. Supplementary Figure S1 outlines the selection process for EHR records included in this study. The EHR record data were used purely to define the UFHPCA boundary.

### State and local data

Socioeconomic vulnerability metrics, digital connectivity, licensed provider metrics, and behavioral data were obtained from FlHealthCHARTS.gov and the County Health Rankings (CHR). County-level birth and perinatal health outcome data were obtained from the Florida Department of Health.^28^ To match the time of the EHR records, rates for each measure were calculated from FL Health Charts' annual rates and weighted by county-level annual population. Counts for each year's numerator and denominator were obtained and aggregated to generate one rate. For CHR data, all data reported are for fiscal years 2011–2017, apart from household computing access data, which were only available for fiscal year 2017.

All data visualizations and tables were created using R statistical software.^29^ FlHealthCHARTS.gov provided rates of computing device access beginning in 2017. The 2021 CHR *z*-scores were obtained for all 67 Florida counties as an established framework to rank each county within Florida from most to least healthy.^30^

### Primary outcomes

Perinatal health outcomes included maternal mortality, neonatal mortality, and preterm birth. Behavioral outcomes included breastfeeding initiation rates and smoking rates during pregnancy. Socioeconomic vulnerability metrics included rates of families with related children younger than 5 years above the poverty line and rates of households with one or more computing devices. Health care access included number of licensed pediatricians, obstetrician gynecologists (OB-GYNs), and family practice physicians.

### Rurality designations

Rurality on the county level was identified according to rurality designations defined by Florida Statutes 288.0656. A county was designated as rural if the population did not exceed 75,000, or if the population did not exceed 125,000 and was contiguous to a county with a population of less than 75,000.^31^

### Analysis

Once counties meeting the threshold to be designated within the UFHPCA were identified, county-level rates of perinatal behaviors, socioeconomic vulnerability metrics, and health care access were compared with state-wide rates. The ggplot2 and grid packages were used to visualize rates geographically by county.^32,33^ County rates were ranked by rates for interpretation. Additionally, risk ratios for rural versus nonrural counties within the UFHPCA were calculated for each measure.

## Results

UF Health Shands Hospital delivered 16,711 infants between June 2011 and April 2017. Of those infants, 112 were unable to be linked to a mother and excluded from the study. Zip code data were missing for a further 43 infants, and the remaining 16,566 infants were linked to a county of residence (Supplementary Fig. S1). Out of 67 counties in the state of Florida, we identified the perinatal catchment area as 13 counties, where ≥5% of infants within the county were delivered within the UF Health system (Table 1). Counties included in the UFHPCA were Alachua, Citrus, Marion, Putnam, Bradford, Columbia, Union, Suwannee, Gilchrist, Levy, Hamilton, Dixie, and Lafayette. The UFHPCA included 3 nonrural counties and 10 rural counties.

**Table 1.** County-Level Demographics of University of Florida Health Perinatal Catchment Area

County	Geographic features			Population demographics (% of population in 2017)					Birth data (June 2011–April 2017)	
	Rurality	Density	Population	NH White	NH Black	NH other	Hispanic	Females 18–35	Live births	Shands births (%)
Alachua	Nonrural	297.0	259,349	62.0	20.0	8.6	9.4	19.4	17,034	42.3
Citrus	Nonrural	243.0	144,922	88.5	2.8	3.3	5.4	6.9	6,161	5.5
Marion	Nonrural	217.0	352,067	71.4	12.5	3.6	12.5	9.3	20,200	18.5
Putnam	Rural	99.6	73,068	71.8	15.9	2.6	9.8	9.5	4,933	10.0
Bradford	Rural	91.1	27,808	74.3	19.0	2.8	3.9	9.2	1,789	26.5
Columbia	Rural	85.9	69,250	72.9	17.8	3.3	6.0	10.0	4,777	13.4
Union	Rural	62.8	15,896	69.4	22.4	2.5	5.7	7.4	961	23.1
Suwannee	Rural	63.6	44,527	75.7	12.9	2.3	9.1	9.0	2,718	16.6
Gilchrist	Rural	49.2	16,977	87.4	4.6	2.4	5.6	9.2	1,131	36.5
Levy	Rural	35.5	40,832	80.1	9.0	2.7	8.3	9.4	2,324	45.9
Hamilton	Rural	27.7	14,749	55.0	33.2	2.8	9.1	7.9	911	16.8
Dixie	Rural	23,2	17,040	84.0	9.6	2.3	4.2	7.7	925	42.5
Lafayette	Rural	16.0	8,651	71.1	14.5	2.1	12.4	8.0	430	29.9

NH, non-Hispanic.

Analysis from the CHR revealed that 12 out of 13 counties in the UFHPCA had a positive health outcomes *z*-score, indicating a high health burden compared with the state rate (Supplementary Fig. S2). According to the Florida Department of Health, the total population of the UFHPCA in 2017 was 1,085,136. During the study period, there were 64,294 live births with 32.5% (20,899) of deliveries coming from rural counties.

The percentage of live births at UF Health Shands Hospital across the UFHPCA ranged from 5.5% in Citrus County to 45.9% in Levy County (Table 1). Alachua, Bradford, Gilchrest, Levy, Dixie, and Lafayette all had ∼30% or more of live births at Shands Hospital.

We found that four counties in our analysis reported no maternal deaths during the study period; however, the remaining nine counties reported age-adjusted maternal death rates that were one to fourfold higher than the statewide rate of 2.1% deaths per 100,000 population (Table 2). With respect to infant mortality, we found that 12 of the 13 counties in the UFHPCA reported mortality rates (6.4–13.1 deaths per 1,000 live births) that were above the state-wide rate (6.1 deaths per 1,000 live births).

**Table 2.** University of Florida Health Perinatal Catchment Area Health Outcomes and Health Behaviors

Area	Perinatal health outcomes			Perinatal behaviors	
	Maternal deaths (age-adjusted rate per 100,000 population)	Infant mortality (per 1,000 live births)	Preterm births (per 1,000 live births)	Births to smoking mothers during pregnancy (%)	Births to mothers who initiate breastfeeding (%)
Florida	2.1	6.1	10.1	6.2	82.9
Alachua	3.0	8.0	10.4	6.8	84.6
Citrus	2.4	7.1	10.1	23.0	74.2
Marion	2.2	8.1	9.0	13.5	74.9
Putnam	5.5	8.0	11.8	20.2	75.3
Bradford	4.8	11.9	11.6	21.9	71.4
Columbia	2.1	9.1	11.0	17.6	68.4
Union	0.0	4.1	13.1	20.9	70.1
Suwannee	0.0	7.9	11.3	16.3	70.4
Gilchrist	0.0	7.0	9.0	15.5	81.4
Levy	3.3	7.3	11.1	18.2	78.6
Hamilton	8.8	10.6	10.5	15.2	54.9
Dixie	9.1	6.4	11.9	24.8	70.1
Lafayette	0.0	6.0	11.9	16.3	73.2

**FIG. 1.**
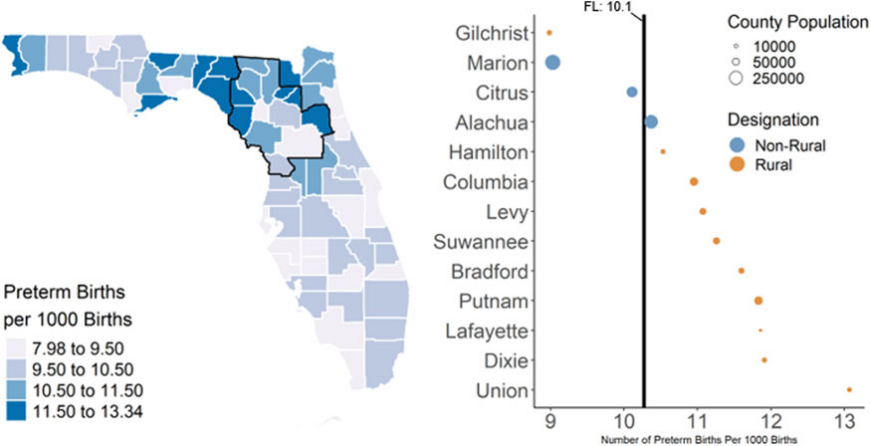
Rates of preterm births in Florida and the UFHPCA. Left—statewide map of preterm birth rates (annualized 2011–2017) with UFHPCA (left; outlined in black). The 13 catchment counties are ordered by rate of preterm births. Right—the statewide rate (10.1% from June 2011 to April 2017) is denoted by the vertical black line. Rates are weighted annually by county population. Ten out of 13 UFHPCA counties had preterm birth rates above the statewide rate, indicating higher levels of health burden within the UFHPCA. UFHPCA, University of Florida Health Perinatal Catchment Area.

**FIG. 2.**
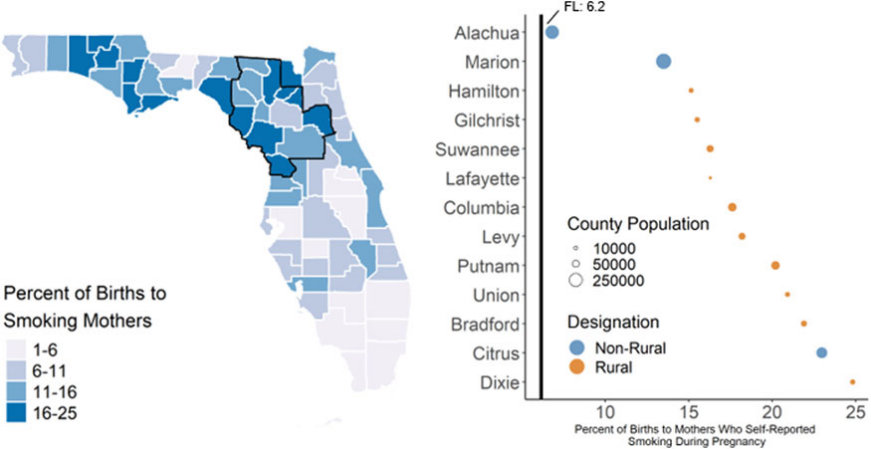
Rates of maternal smoking during pregnancy in Florida and the UFHPCA. Left—statewide map of the percent of births (annualized 2011–2017) to mothers who reported smoking during pregnancy. UFHPCA outlined in black. Right—the 13 catchment counties ordered by maternal smoking rate, the statewide rate (6.2% between June 2011 and April 2017) is denoted by the vertical black line. Rates are weighted annually by county population. All 13 counties in the UFHPCA had maternal smoking rates during pregnancy above the statewide rate.

**FIG. 3.**
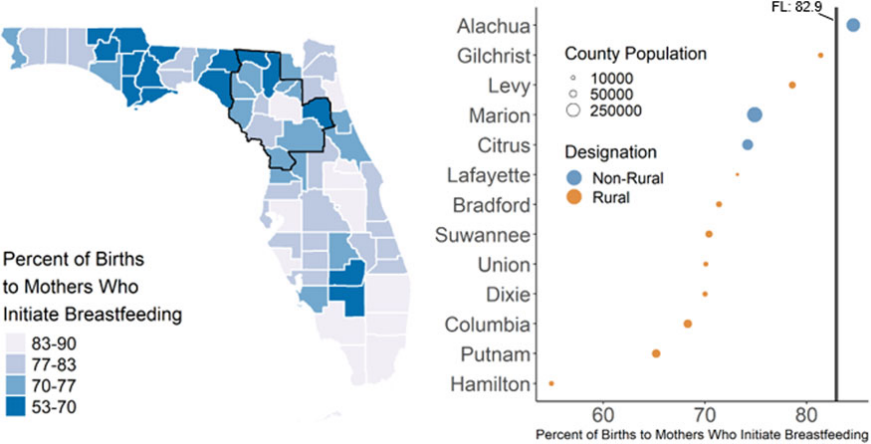
Rates of breastfeeding initiation in Florida and the UFHPCA. Left—statewide map of county-level breastfeeding initiation rates (annualized 2011–2017) with the UFHPCA (left; outlined in black). Right—the 13 catchment counties ordered by breastfeeding initiation rate where the statewide rate (82.9% between June 2011 and April 2017) is denoted by the vertical black line (right). Rates are weighted by county-level annual population. Within the catchment, all counties, except Alachua County, had rates below the statewide rate, indicating higher levels of health burden within the UFHPCA.

We also found that preterm birth rates (Fig. 1, 9.0–13.1 preterm infants per 1,000 live births) among the UFHPCA counties were above the state-wide rate of 10.1 infants per 1,000 live births, except for Marion, Citrus, and Gilchrist counties, which had rates of 9.0, 10.1, and 9.0, respectively. All counties in the UFHPCA had maternal smoking rates (Fig. 2, 6.8%–24.8%) above the statewide rate (6.2%). Within the UFHPCA, only Alachua County (84.3%) had a higher breastfeeding initiation rate than the statewide rate (82.9%), with the remaining catchment counties' rates ranging from 52.8% to 81.4% (Fig. 3).

**FIG. 4.**
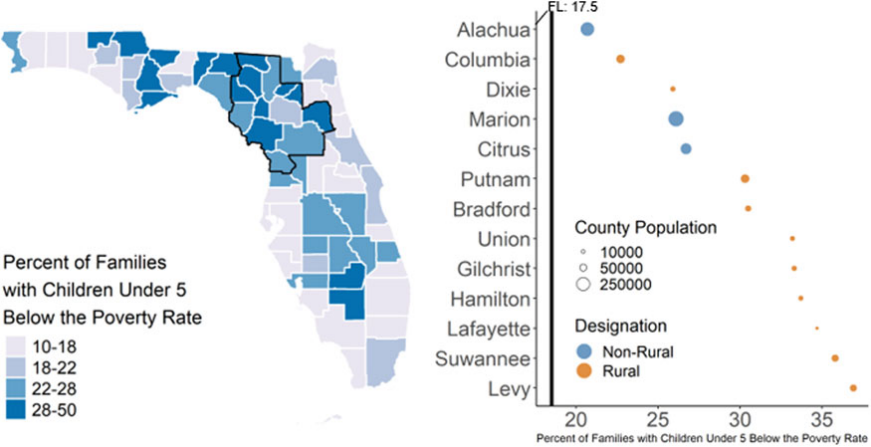
Poverty rate for families with children under five in Florida and the UFHPCA. Left—statewide map of families with related children under the age of five living below the poverty rate (annualized 2011–2017) with the UFHPCA (left; outlined in black). Right—the 13 catchment counties are ordered by rate where the statewide rate (17.5% from June 2011 to April 2017) is denoted by the vertical black line (right). Rates are weighted by county-level annual population. Within the UFHPCA, all counties had poverty rates above the statewide rate, indicating higher levels of health burden within the UFHPCA.

**FIG. 5.**
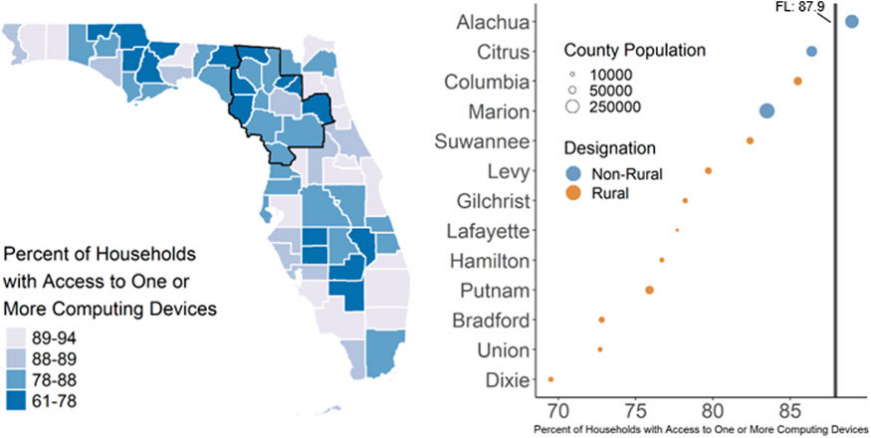
Rates of access to household computing devices in Florida and the UFHPCA in 2017. Left—statewide map of rates of computing device access within households (2017) in the UFHPCA (left; outlined in black). Right—the 13 catchment counties are ordered by household rates of access to one or more computing device, where the 2017 statewide rate (87.9%) is denoted by the vertical black line (right). Within the UFHPCA all counties, except Alachua County, had computing device access rates below the statewide rate, indicating higher levels of health burden within the UFHPCA.

All counties other than Columbia (17.6%) and Suwanee (16.3%) in the catchment had poverty rates that exceeded the statewide rate of 18.5% for families with young children living in poverty (Table 3, Fig. 4, 20.7%–36.9%). All counties other than Alachua County (89.0%) in the UFHPCA had lower rates of access to computing devices in 2017 than the statewide rate (87.9%) (Fig. 5, 60.5%–86.4%). Three rural counties with the lowest access to computing devices in 2017 were Union (72.7%), Bradford (72.8%), and Dixie (69.5%) counties. Seven rural counties in the UFHPCA did not have a licensed OB-GYN in the entire county. Twelve out of 13 counties in the UFHPCA were below the statewide rate for at least one type of provider during the period studied. For the 13 counties in the UFHPCA, 10 had fewer than 50% of the state average density for licensed pediatricians, 7 had fewer than 50% of the state average density for family practice physicians, and 9 had fewer than 50% of the state average density for OB-GYNs.

**Table 3.** University of Florida Health Perinatal Catchment Area Socioeconomic Vulnerability and Health Care Access

Area	Socioeconomic vulnerability (%)		Licensed providers available (per 100,000 population)		
	Families with Related children (<5 years) below the poverty line	Households with one or more computing devices	Pediatricians	Family practice physicians	OB-GYNs
Florida	17.5	87.9	19.6	21.4	9.7
Alachua	20.7	89.0	64.6	45.8	21.4
Citrus	26.7	86.4	7.0	21.5	4.0
Marion	26.1	83.5	8.2	18.1	4.8
Putnam	30.3	75.9	8.5	11.2	9.1
Bradford	21.9	72.8	1.8	15.2	0.6
Columbia	17.6	85.5	5.9	15.7	2.0
Union	20.9	72.7	1.1	8.5	0.0
Suwannee	16.3	82.4	0.4	3.8	0.0
Gilchrist	33.3	78.2	6.9	5.9	0.0
Levy	36.9	79.7	1.7	13.2	0.0
Hamilton	33.7	76.7	0.0	2.3	0.0
Dixie	24.8	69.5	4.1	7.1	0.0
Lafayette	34.7	77.7	0.0	1.9	0.0

OB-GYNs, obstetrician gynecologists.

**FIG. 6.**
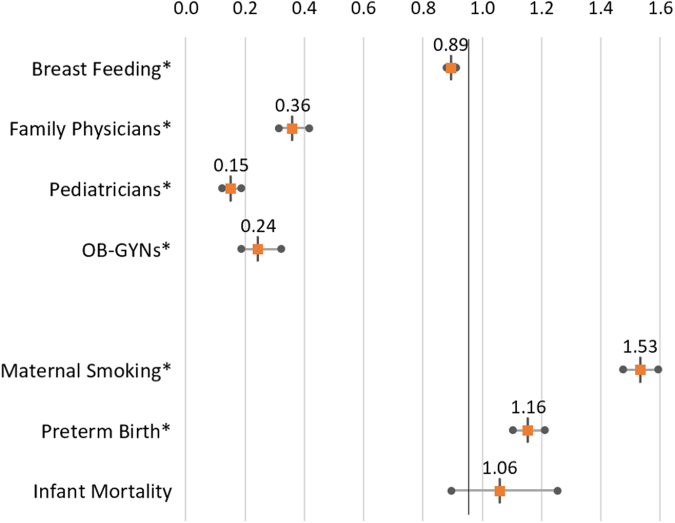
Rural versus nonrural risk ratios, 2011–2017. Plot of rate ratios and CIs for comparing the health behaviors, health care providers, and health outcomes between the rural and nonrural counties in the UFHPCA. LCL and UCL given for significance of *p* ≤ 0.05 and * marks statistically significant disparity. Rates of intent to breastfeed and all three physician types examined were significantly lower in the rural counties than in the nonrural counties, whereas rates of maternal smoking and preterm birth were significantly higher in rural counties than in nonrural counties. CI, confidence interval; LCL, lower confidence interval; UCL, upper confidence interval.

We also compared the relative risk ratios between rural and nonrural counties in the UFHPCA for the same measures (Table 4, Fig. 6). Breastfeeding initiation of rural counties were 89% of their nonrural counterparts. Family physicians' risk ratio was significantly lower in rural counties, with counts of family physicians only 36% of counts in nonrural counties. A similar trend was found for both pediatricians (15%) and OB-GYNs (24%). Maternal smoking rates were 53% higher in rural counties, and preterm birth 16% higher. Infant mortality and maternal deaths lacked significant *p*-values, due to the relative rarity of both occurrences which limited the usefulness of risk ratios between rural and nonrural counties.

**Table 4.** Rural Versus Nonrural Risk Ratios, 2011–2017

	Risk ratio	Upper confidence level	Lower confidence level
	Open	High	Low
Breastfeeding initiation^*^	0.89	0.91	0.88
Family physicians^*^	0.36	0.42	0.31
Pediatricians^*^	0.15	0.19	0.12
OB-GYNs^*^	0.24	0.31	0.19
Maternal smoking^*^	1.53	1.59	1.48
Preterm births^*^	1.16	1.21	1.10
Infant mortality	1.06	1.25	0.90
Maternal deaths	1.04	2.78	0.39

^*^ Indicates significance at 0.05 level.

## Discussion

### Summary

Geographic variation in health outcomes has consistently demonstrated that rural communities are disproportionately affected^34–37^ in the domains of mortality and clinical care.^38^ This study identified 13 Florida counties as part of the UFHPCA, which included rural and nonrural counties, representing more than 64,000 deliveries over nearly 5 years. Twelve out of 13 counties in the UFHPCA had a higher health burden compared with the state rate, while 3 UFHPCA counties were in the top 5 Florida counties for overall health burden. Collectively, our results revealed that relative to statewide averages, north central Florida is characterized by both rural and nonrural counties with higher maternal and infant mortality and higher levels of smoking during pregnancy and childhood poverty and lower levels of breastfeeding initiation.

### Health care access

In 2014, less than half of all rural counties in the United States had hospital-based obstetric care,^39^ emphasizing the impact of socioeconomic status and transport resources^40^ on adequate health care services for rural populations. An important observation made by this study is that 7 out of 13 counties in our analysis did not have licensed OB-GYNs, suggesting that patients living in these areas may reside within a perinatal health care desert.

Women living in perinatal health care deserts experience much higher rates of adverse outcomes during pregnancy and childbirth compared with those living outside these areas.^41^ Therefore, many of the adverse perinatal outcomes identified in this article are likely correlated with the lack of key health care providers in local counties, forcing pregnant women to seek care in a different health system. In Louisiana from 2016 to 2017, women living in perinatal health care deserts experienced a 91% increase in risk of maternal mortality during pregnancy and up to 1 year postpartum, regardless of the women's age, race or ethnicity, or socioeconomic status.^42^

Our results were generally consistent with these observations, whereby maternal mortality was the highest in counties without licensed OB-GYNs. Given the complexity of health care accessibility in rural communities,^43,44^ our results highlight the importance of defining “high-risk” areas that can inform precision public health interventions focused on reducing maternal mortality in rural communities.^45^ Having a single health catchment that serves these communities provides researchers with the data to identify counties, and even neighborhoods that need the most immediate influx in health care services.

### Maternal mortality

The United States consistently ranks last in maternal mortality among industrialized nations, with ∼700 women each year dying due to pregnancy or delivery complications.^2^ Kozhimannil et al found that rural residents had a 9% greater probability of severe maternal morbidity and mortality during pregnancy, compared with urban residents.^10^ Moreover, estimates suggest that nearly three in five pregnancy-related deaths in the United States are potentially preventable and this proportion can vary, with some cases indicating that up to 90% of maternal deaths may be preventable.^8,46,47^ We found that four counties in our analysis reported no maternal deaths during the period studied; however, the remaining nine counties reported age-adjusted maternal death rates that were one to fourfold higher than the statewide rate of 2.1% deaths per 100,000 population. Previous work in Florida has demonstrated that overall public health expenditures were not directly associated with improvements perinatal health outcomes, such as maternal deaths.^48^

Specifically, Bernet et al reported that pregnancy-related public health programs across all 67 Florida counties resulted in statistically significant reductions in high-risk groups that included the black maternal death rate and the black/white maternal death gap after adjusting for income, employment, and access to care.^48^ Although these analyses did not consider rurality, clinical efforts to reduce maternal deaths in rural communities include the development of evidence-based clinical practices called maternal safety bundles that are designed to reduce the incidence of and risks associated with severe maternal morbidity and mortality.^10^ Taken together, public health policies and clinical practices that aim to address maternal mortality should ensure the representation of rural residents within decision-making bodies and public health interventions.^49^

### Health behaviors

Maternal health behaviors during pregnancy and delivery are key determinants of infant outcomes. Our study revealed that all counties in the UFHPCA had maternal smoking rates that were higher than the statewide average. Prenatal smoking increases risks of adverse pregnancy outcomes, including stillbirth,^50^ preterm birth,^51^ and infant mortality.^52^ We also found that every county in the UFHPCA, except Alachua County had lower breastfeeding initiation rates than the statewide average. As advised in the Center for Disease Control's Breastfeeding Report Card, 2020, individualized support in the first few hours and days is critical to help mothers meet their breastfeeding goals.

Our analysis also revealed that rural counties generally reported neonatal mortality and preterm birth rates that were higher than the statewide averages. Family poverty, another SDoH, influences many aspects of perinatal health, including maternal diet and exercise and access to perinatal care. Poverty is one component of socioeconomic status, which has also been intimately associated with birthweight.^53^ Birthweight is tied to many outcomes during the perinatal period and long-term health.^54^ Higher poverty is associated with a lower birthweight.^55^

Our study revealed that every county in the UFHPCA exceeded the Florida statewide rate of poverty. Taken together, our results demonstrate that UFHPCA counties had some of the lowest breastfeeding initiation rates across the state of Florida, which highlights the need for innovative public health interventions that promote breastfeeding in rural and nonrural areas. For example, a meta-analysis identified smoking as an important factor for breastfeeding, where nonsmoking mothers had higher breastfeeding initiation and continuation rates.^56^ Recognizing that many of the counties in the UFHPCA had both higher maternal smoking rates and lower breastfeeding initiation rates, an intervention that aims to lower smoking rates could improve both smoking and breastfeeding behaviors.

### Digital connectivity

With the increasing utilization of telemedicine throughout the United States, many states have begun integrating telemedicine into obstetrics and maternal health clinics. Access to telemedicine services could be an effective technique to improve maternal health outcomes for at-risk populations in rural areas, such as many counties within the UFHPCA. However, access to telemedicine is only available through a computing device with digital connectivity capability.

Measuring computing device access throughout the UFHPCA provides a more accurate understanding of patients' abilities to utilize telemedicine. Long driving times to reach physicians and limited options for perinatal care and digital information in rural counties may be detrimental to maternal health outcomes.^41^ In 2011, The Georgia Department of Public Health partnered with health districts and maternal medicine specialists to provide telemedicine consultations for African American and Hispanic women with high-risk pregnancies. In an 18-month period, the preterm labor rate was reduced in the target population from 18.8% to 8% across 500 pregnancies.

Additionally, there was a noticeable increase in the adherence to follow-up care following delivery.^57^ Telemedicine services allowed patients to communicate with their physicians *via* computers, phones, or tablets.^58^ Health interventions that leverage telemedicine have been associated with improvements in obstetrics outcomes.^59^ The number of maternity deserts in the UFHPCA highlights the need for innovative maternity care that expands beyond a single hospital.

### Strengths and limitations

The UFHPCA defines a region of North Central Florida. The strengths of using county rankings as a primary metric for comparison include ease of interpretation and visualization of unmet medical needs that can help generate active stakeholder participation in appropriate public health and policy actions. Because data are systematically collected through existing state and federal agencies, the broader impact of interventions can be readily assessed with new data pulls. However, using existing county-level data limits the resolution of data that can be achieved. For example, while the available data allow us to count the number of licensed OB-GYNs by county, it prevents us from capturing obstetric care offered by other providers (*e.g.,* family medicine physicians who provide obstetric care, nurse practitioners, or midwives). Likewise, although we were able to compare rural and nonrural counties, such a binary designation may not capture local neighborhood effects.

Given that the socioeconomic heterogeneity of rural areas has been documented,^60^ future studies on the UFHPCA should look beyond the binary rurality designation of each county and examine health outcomes related to poverty and other neighborhood demographic characteristics using census tract-level investigations. Additionally, the only perinatal health behaviors measured were maternal smoking and breastfeeding initiation rates. Future studies could include a more comprehensive list of behaviors to provide a more descriptive picture.

A higher resolution understanding of perinatal health outcomes and locations of perinatal care providers within the UFHPCA enhances understanding of the areas with the highest perinatal health burden and subsequently can identify areas where public health initiatives would provide the most benefit. Alachua, location of UF Health Shands Hospital at the UF, is the only county in the UFHPCA that is above the Florida rate for average numbers of all three health care providers measured. Future analysis should investigate not only how hospitals within a single catchment area provide care for surrounding counties but how this influx of nonlocal patients affects hospital resources and provider capability.

## Conclusions

The results of this study highlight the importance of understanding the effects of SDoH on health care accessibility and perinatal outcomes in both rural and nonrural counties within an accurate service catchment area. Delineating the boundaries of the UFHPCA allows researchers and providers to focus primary data collection and intervention implementation within an actionable region, facilitating collaborations with the appropriate nonprofits and policymakers to work toward the goal of addressing health disparities and communication inequalities. This article therefore represents an essential first step toward launching interventions to address the impact of SDoH on perinatal health. These findings provide additional insight into prioritizing strategies that address SDoH and expand health care access to rural counties, such as improving health care access *via* telemedicine. By understanding how health behaviors and socioeconomic factors juxtapose with the physical environments in which patients live (*i.e.,* rurality), the findings help identify potential policies and programs that may improve perinatal outcomes.

## Supplementary Material

Supplemental data

Supplemental data

## Data Availability

EHR data used in this study cannot be shared publicly to protect participant confidentiality. All requests to review data that may include potential subject and/or patient identifiers will be reviewed by our University's Privacy Office and Institutional Review Board to ensure adequate protection of subjects. Any dissemination approved by the University will be reviewed by a University honest data broker to ensure appropriate legal and ethical compliance with regulatory agencies. Requests for data should be addressed to Chris Harle at the UF (charle@ufl.edu). County-level data on live birth rates, perinatal health data, and socioeconomic vulnerability metrics are publicly available from the Florida Department of Health's FL Health Charts (https://www.flhealthcharts.gov/Charts/default.aspx). The CHR's Health Outcome z-scores are also publicly available (https://www.countyhealthrankings.org/explore-health-rankings/use-data). The R code used to create all maps and tables displayed in this study is available in the GitHub repo “mombaby-ehr-SdoH” (https://github.com/lemaslab/mombaby-ehr-SdoH).

## Abbreviations Used

CHR: County Health Rankings
CI: confidence interval
EHR: electronic health records
ER: emergency room
LCL: lower confidence interval
NH: non-Hispanic
OB-GYN: obstetricians and gynecologists
SDoH: social determinants of health
UCL: upper confidence interval
UF: University of Florida
UFHPCA: University of Florida Health Perinatal Catchment Area
