# Supplementary figures and images for: Perinatal Health Outcomes Across Rural and Nonrural Counties Within a Single Health System Catchment
Source: Womens Health Rep (New Rochelle). 2023 Apr 17;4(1):169–81. doi: 10.1089/whr.2022.0061 (PMC10122232; doi:10.1089/whr.2022.0061)

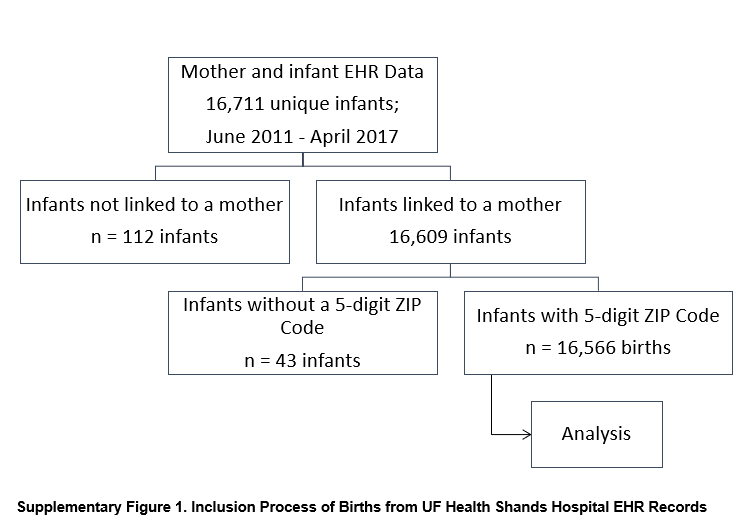

Supplement: Supplemental data [file Suppl_FigS1.PNG]

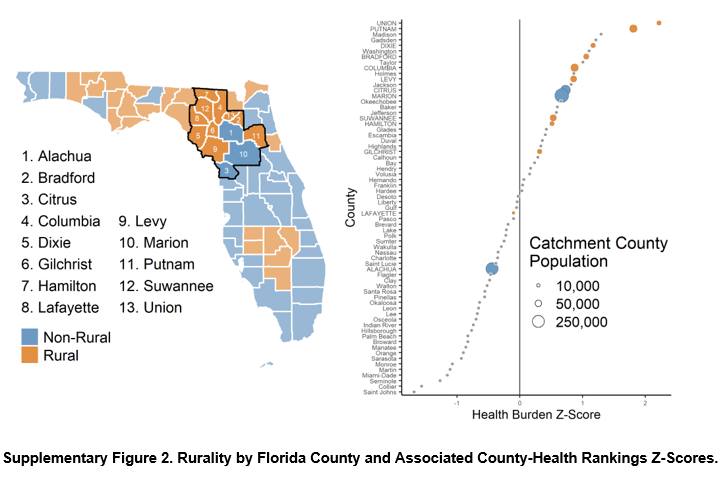

Supplement: Supplemental data [file Suppl_FigS2.png]
